# Impact of *agr* Functionality on the Outcome of Patients with Methicillin-Susceptible Staphylococcus aureus Bacteremia

**DOI:** 10.1128/spectrum.00116-21

**Published:** 2021-08-11

**Authors:** Jeong Eun Lee, Shinwon Lee, Sohee Park, Soon O. Lee, Sun H. Lee

**Affiliations:** a Department of Internal Medicine, Pusan National University School of Medicine and Medical Research Institute, Pusan National University Hospital, Busan, Republic of Korea; University of Pittsburgh School of Medicine

**Keywords:** MSSA, *Staphylococcus aureus*, *agr*, bacteremia, quorum sensing

## Abstract

Dysfunctional accessory gene regulator (*agr*) is associated with unfavorable outcomes in invasive methicillin-resistant Staphylococcus aureus infections. However, it is unknown whether this association persists in methicillin-susceptible Staphylococcus aureus bacteremia (MSSA-B). This study evaluated the association between *agr* dysfunction and mortality in patients with MSSA-B. This retrospective cohort study included MSSA-B patients (≥15 years) enrolled from June 2014 to June 2019 and retrospectively collected their demographic and clinical information. Stored causative strains were measured for *agr* functionality by δ-hemolysin production assays. Among 244 MSSA-B patients, 91 (37.3%) and 153 (62.7%) had dysfunctional and functional *agr* MSSA-B, respectively. Ninety-day mortality occurred in 18.7% and 17.6% dysfunctional and functional groups, respectively (*P* = 0.97). Kaplan-Meier analysis showed that mortality due to dysfunctional *agr* MSSA-B was not significantly higher (*P* = 0.82). Age, sites, the severity of infection, and comorbidity adjusted hazard ratio (aHR) of the dysfunctional group for 90-day mortality was 1.303 (95% confidence interval [CI], 0.698 to 2.436, *P* = 0.41). Mortality due to MSSA-B with sequential organ failure assessment (SOFA) scores of 2 to 5 was significantly higher in the dysfunctional group (*P* = 0.03), and the dysfunctional *agr* aHR for 90-day mortality was 3.260 (95% CI, 1.050 to 10.118, *P* = 0.04). The *agr* dysfunction of causative organisms can have a significant effect on the outcomes of MSSA-B in patients with moderate severity (SOFA scores 2 to 5).

**IMPORTANCE** Few studies have examined the association between methicillin-susceptible Staphylococcus aureus (MSSA) infection and accessory gene regulator (*agr*) functionality. We evaluated the association between *agr* dysfunction and mortality in patients with MSSA bacteremia. Dysfunctional *agr* is associated with lower survival in MSSA bacteremia patients with moderately severe sequential organ failure assessment (SOFA) scores of 2 to 5. We found that the *agr* functionality of causative organisms may have an effect on patients’ outcomes in MSSA like in methicillin-resistant S. aureus.

## INTRODUCTION

Staphylococcus aureus is a common and important cause of hospital-acquired and community-acquired bloodstream infection with high morbidity and mortality, despite prompt control of the source and appropriate antimicrobial therapy (1–3). S. aureus produces potential virulence factors, such as toxins, degradative enzymes, antimicrobial resistance genes, and immune evasion mechanisms, which contribute to pathogenicity and the ability to colonize the host (3, 4). Quorum sensing, which is a population density-dependent and environment-dependent gene regulation, is an important mechanism for the control of these virulence factors (5). The accessory gene regulator (*agr*) locus of S. aureus is responsible for quorum sensing and coordinates the expression of various virulence factors (6). The *agr* quorum-sensing system leads to the spread of infection by dispersion of the biofilm and increased production of exoproteins and murein hydrolases at high cell density (7, 8). Loss of *agr* activity has been associated with enhanced biofilm formation and decreased autolysis rates, which can contribute to the persistence of infection and poor outcomes (4).

Many clinical studies have demonstrated that a considerable proportion of invasive methicillin-resistant S. aureus (MRSA) infection is caused by the strain with dysfunctional *agr* (9–11).

Dysfunctional *agr* in invasive MRSA infection was associated with overall unfavorable outcomes, such as persistent bacteremia and high in-hospital mortality rates (10).

Dysfunctional *agr* is not uncommon even in invasive methicillin-susceptible S. aureus (MSSA) infections. However, few studies have examined the association between the outcomes of invasive MSSA infection and *agr* functionality, and the results of such studies are inconclusive (10).

The objective of this study was to evaluate the association between *agr* dysfunction and mortality in patients with MSSA bacteremia (MSSA-B), adjusting for important confounding factors, such as comorbid conditions and severity of the illness.

## RESULTS

A total of 244 patients with MSSA-B were enrolled during the study period. The overall median age of the enrolled patients was 66.5 (interquartile range [IQR], 56 to 74) years, and 68.8% of these patients were males. Among the MSSA-B patients, 63.9% had more than one comorbidity, and the median Charlson comorbidity weighted index (CCWI) score was 4 (IQR, 2 to 6). The prevalence of community-onset bacteremia was 60.7%, and the lung (17.6%) was the most common site of infection, followed by the skin and soft tissues (16.8%). The median sequential organ failure assessment (SOFA) score of the patients was 3 (IQR, 1 to 5), and 73.4% of the patients had a SOFA score of ≥2.

Among the 244 MSSA-B patients, 91 (37.3%) were infected by MSSA with dysfunctional *agr* (the dysfunctional *agr* group) and 153 (62.7%) were infected by MSSA with functional *agr* (the functional *agr* group; Table 1). There were no significant differences in demographics and clinical manifestations between the dysfunctional and functional *agr* groups, although the dysfunctional *agr* group tended to have an initial severity lower than that of the functional *agr* group (SOFA score 0 to 1 [33.0% versus 22.9%], 2 to 5 [47.3% versus 50.3%], ≥6 [19.8% versus 26.8%], *P* = 0.18). More than 98% of patients in both groups received appropriate empirical antibiotics, and there was no significant difference in the rate of use of definitive antibiotics between the groups (Table 1). There was no significant difference in the 7-day, 30-day, and 90-day mortality rates between the dysfunctional and functional *agr* groups (Table 1).

**TABLE 1:** Comparison of demographic, clinical, and microbiological characteristics according to the functionality of *agr* locus of MSSA bacteremia^*a*^

Characteristic	Value for group		*P* value
	Functional *agr* (*n* = 153)	Dysfunctional *agr* (*n* = 91)	
Median age, yrs (IQR)	65 (56–74)	67.0 (56–74.5)	0.447
Older age (≥65 yrs)	78 (51.0%)	52 (57.1%)	0.424
Male	106 (69.3%)	62 (68.1%)	0.964
Community-onset	92 (60.1%)	56 (61.5%)	0.934
Comorbidity			
Cardiovascular disease	16 (10.5%)	8 (8.8%)	0.841
Central nervous disease	23 (15.0%)	11 (12.1%)	0.652
Chronic lung disease	2 (1.3%)	1 (1.1%)	>0.999
Chronic liver disease	12 (7.8%)	5 (5.5%)	0.662
Chronic kidney disease	18 (11.8%)	10 (11.0%)	>0.999
Connective tissue disease	3 (2.0%)	2 (2.2%)	>0.999
Solid organ malignancy	22 (14.4%)	14 (15.4%)	0.978
Hematologic malignancy	17 (11.1%)	5 (5.5%)	0.211
Diabetes mellitus	42 (27.5%)	25 (27.5%)	>0.999
HIV infection	1 (0.7%)	0 (0.0%)	>0.999
Median CCWI, score (IQR)	4 (2–6)	3 (2–5.5)	0.883
High CCWI (CCWI ≥ 3)	102 (66.7%)	61 (67.0%)	>0.999
Site of infection			
Unknown	34 (22.2%)	16 (17.6%)	0.481
Central line infections	22 (14.4%)	16 (17.6%)	0.628
Infective endocarditis	5 (3.3%)	4 (4.4%)	0.920
Osteomyelitis	24 (15.7%)	17 (18.7%)	0.669
Septic arthritis	5 (3.3%)	6 (6.6%)	0.373
Skin and soft tissue	30 (19.6%)	11 (12.1%)	0.180
Lung	28 (18.3%)	15 (16.5%)	0.852
Combined deep-seated abscesses	26 (17.0%)	21 (23.1%)	0.319
*agr* genotype			0.001
I	106 (69.3%)	54 (59.3%)	
II	15 (9.8%)	5 (5.5%)	
III	19 (12.4%)	29 (31.9%)	
IV	13 (8.5%)	3 (3.3%)	
*blaZ* genotype			0.009
A	16 (10.5%)	17 (18.7%)	
B	40 (26.1%)	34 (37.4%)	
C	60 (39.2%)	18 (19.8%)	
D	4 (2.6%)	5 (5.5%)	
None	33 (21.6%)	17 (18.7%)	
Cefazolin inoculum effect	13 (8.5%)	12 (13.2%)	0.342
Vancomycin MIC			0.321
<0.5 mg/liter	76 (49.7%)	42 (46.2%)	
1 mg/liter	74 (48.4%)	49 (53.8%)	
2 mg/liter	3 (2.0%)	0 (0.0%)	
SOFA score			0.178
0–1	35 (22.9%)	30 (33.0%)	
2–5	77 (50.3%)	43 (47.3%)	
≥6	41 (26.8%)	18 (19.8%)	
Appropriate empirical antibiotics	150 (98.0%)	91 (100%)	0.457
Definitive antibiotics			0.925
Nafcillin	54 (35.3%)	31 (34.1%)	
Cefazolin	39 (25.5%)	22 (24.2%)	
Others	60 (39.2%)	38 (41.8%)	
Outcomes			
Median duration of bacteremia, day (IQR)	1 (1–4)	1 (1–3)	0.267
Persistent bacteremia	22 (14.4%)	9 (9.9%)	0.413
7-D death	10 (6.5%)	8 (8.8%)	0.690
30-D death	22 (14.4%)	14 (15.4%)	0.978
90-D death	27 (17.6%)	17 (18.7%)	0.975

a *agr*, accessory gene regulator; MSSA, methicillin-susceptible Staphylococcus aureus; IQR, interquartile range; HIV, human immunodeficiency virus; CCWI, Charlson comorbidity weighted index; SOFA, sequential organ failure assessment; D, day.

The 90-day mortality rate of MSSA-B was 18% (44 of 244 patients). In the bivariate analysis, a high CCWI (CCWI ≥ 3), a high SOFA score, sites of infection of endocarditis or pneumonia, and use of drugs other than nafcillin or cefazolin as definitive antibiotics were more likely to be associated with 90-day mortality, whereas community-onset bacteremia was less likely to be associated with the 90-day mortality (Table 2). In the logistic regression analysis, a high CCWI, a high SOFA score, and infective endocarditis were independently associated with mortality, whereas community-onset infection was negatively associated with mortality (Table 3).

**TABLE 2:** Bivariate analysis of risk factors for 90-day mortality among patients with MSSA bacteremia^*a*^

Characteristic	Value for group		*P* value
	Survived (*n* = 200)	90-day mortality (*n* = 44)	
Median age, yrs (IQR)	66 (54.5–74)	68 (56.5–75.5)	0.23
Older age (≥65 yrs)	105 (52.5%)	25 (56.8%)	0.72
Male	135 (67.5%)	33 (75.0%)	0.43
Community onset	129 (64.5%)	19 (43.2%)	0.01
Comorbidity			
Cardiovascular disease	19 (9.5%)	5 (11.4%)	0.92
Central nervous disease	28 (14.0%)	6 (13.6%)	>0.999
Chronic lung disease	3 (1.5%)	0 (0.0%)	0.95
Chronic liver disease	11 (5.5%)	6 (13.6%)	0.11
Chronic kidney disease	23 (11.5%)	5 (11.4%)	>0.999
Connective tissue disease	3 (1.5%)	2 (4.5%)	0.48
Solid organ malignancy	26 (13.0%)	10 (22.7%)	0.16
Hematologic malignancy	16 (8.0%)	6 (13.6%)	0.37
Diabetes mellitus	56 (28.0%)	11 (25.0%)	0.83
HIV infection	1 (0.5%)	0 (0.0%)	>0.999
Median CCWI, score (IQR)	3 (2–5)	4 (3–7)	0.003
High CCWI (CCWI ≥ 3)	124 (62.0%)	39 (88.6%)	0.001
Site of infection			
Unknown	37 (18.5%)	13 (29.5%)	0.15
Central line infections	33 (16.5%)	5 (11.4%)	0.53
Infective endocarditis	5 (2.5%)	4 (9.1%)	0.10
Osteomyelitis	37 (18.5%)	4 (9.1%)	0.20
Septic arthritis	9 (4.5%)	2 (4.5%)	>0.999
Skin and soft tissue infection	38 (19.0%)	3 (6.8%)	0.08
Pneumonia	29 (14.5%)	14 (31.8%)	0.01
Combined deep-seated abscesses	40 (20.0%)	7 (15.9%)	0.68
*agr* genotype			0.43
I	129 (64.5%)	31 (70.5%)	
II	15 (7.5%)	5 (11.4%)	
III	43 (21.5%)	5 (11.4%)	
IV	13 (6.5%)	3 (6.8%)	
Dysfunctional *agr* locus	74 (37.0%)	17 (38.6%)	0.97
*blaZ* genotype			0.664
A	29 (14.5%)	4 (9.1%)	
B	57 (28.5%)	17 (38.6%)	
C	65 (32.5%)	13 (29.5%)	
D	8 (4.0%)	1 (2.3%)	
None	41 (20.5%)	9 (20.5%)	
Cefazolin inoculum effect	22 (11.0%)	3 (6.8%)	0.58
Vancomycin MIC			0.626
<0.5 mg/liter	98 (49.0%)	20 (45.5%)	
1 mg/liter	99 (49.5%)	24 (54.5%)	
2 mg/liter	3 (1.5%)	0 (0.0%)	
SOFA score			<0.001
0–1	64 (32.0%)	1 (2.3%)	
2–5	105 (52.5%)	15 (34.1%)	
≥6	31 (15.5%)	28 (63.6%)	
Appropriate empirical antibiotics	197 (98.5%)	44 (100.0%)	0.95
Definitive antibiotics			0.04
Nafcillin	73 (36.5%)	12 (27.3%)	
Cefazolin	54 (27.0%)	7 (15.9%)	
Others	73 (36.5%)	25 (56.8%)	

a MSSA, methicillin-susceptible Staphylococcus aureus; IQR, interquartile range; HIV, human immunodeficiency virus; CCWI, Charlson comorbidity weighted index; *agr*, accessory gene regulator; SOFA, sequential organ failure assessment.

**TABLE 3:** Multivariate logistic regression analysis of risk factors for 90-day mortality among patients with MSSA bacteremia^*a*^

Characteristic	aOR (95% CI)	*P* value
Dysfunctional *agr*	1.558 (0.669–3.653)	0.30
Cefazolin inoculum effect	0.802 (0.141–3.402)	0.78
Community-onset bacteremia	0.366 (0.147–0.870)	0.03
High CCWI (CCWI ≥ 3)	6.260 (1.807–25.156)	0.006
SOFA score		
0–1	1	
2–5	8.0055 (1.453–150.494)	0.05
≥6	52.276 (8.943–1,013.780)	<0.001
Infective endocarditis	8.131 (1.260–56.753)	0.03
Definitive antibiotics		
Nafcillin	1	
Cefazolin	0.929 (0.242–3.395)	0.91
Others	1.443 (0.462–4.780)	0.44

a MSSA, methicillin-susceptible Staphylococcus aureus; aOR, adjusted odds ratio; CI, confidence interval; *agr*, accessory gene regulator; CCWI, Charlson comorbidity weighted index; SOFA, sequential organ failure assessment.

Ninety-day mortality rate was 1.5% (1 of 65 patients), 12.5% (15 of 120 patients), and 47.5% (28 of 59 patients) at SOFA scores of 0 to 1, 2 to 5, and ≥6, respectively. There was a tendency for MSSA-B in the dysfunctional *agr* group to have a mortality rate higher than that in the functional *agr* group at SOFA scores of 2 to 5, although there was no statistically significant difference between the two groups in the severity subgroup analysis (Table 4).

**TABLE 4:** Mortality due to MSSA bacteremia stratified by the initial severity of infections between functional and dysfunctional *agr* groups^*a*^

	Mortality rate for group								
	SOFA 0–1			SOFA 2–5			SOFA ≥6		
Time of mortality (day)	Fx *agr* (*n* = 35)	DysFx *agr* (*n* = 30)	*P* value	Fx *agr* (*n* = 77)	DysFx *agr* (*n* = 43)	*P* value	Fx *agr* (*n* = 41)	DysFx *agr* (*n* = 18)	*P* value
7	0 (0%)	0 (0%)		0 (0%)	4 (9.3%)	0.03	10 (24.4%)	4 (22.2%)	>0.999
30	0 (0%)	0 (0%)		5 (6.5%)	7 (16.3%)	0.16	17 (41.5%)	7 (38.9%)	>0.999
90	1 (2.9%)	0 (0%)	>0.999	6 (7.8%)	9 (20.9%)	0.07	20 (48.8%)	8 (44.4%)	0.98

a MSSA, methicillin-susceptible Staphylococcus aureus; SOFA, sequential organ failure assessment; Fx *agr*, functional accessory gene regulator; DysFx *agr*, dysfunctional accessory gene regulator; D, day.

**FIG 1:**
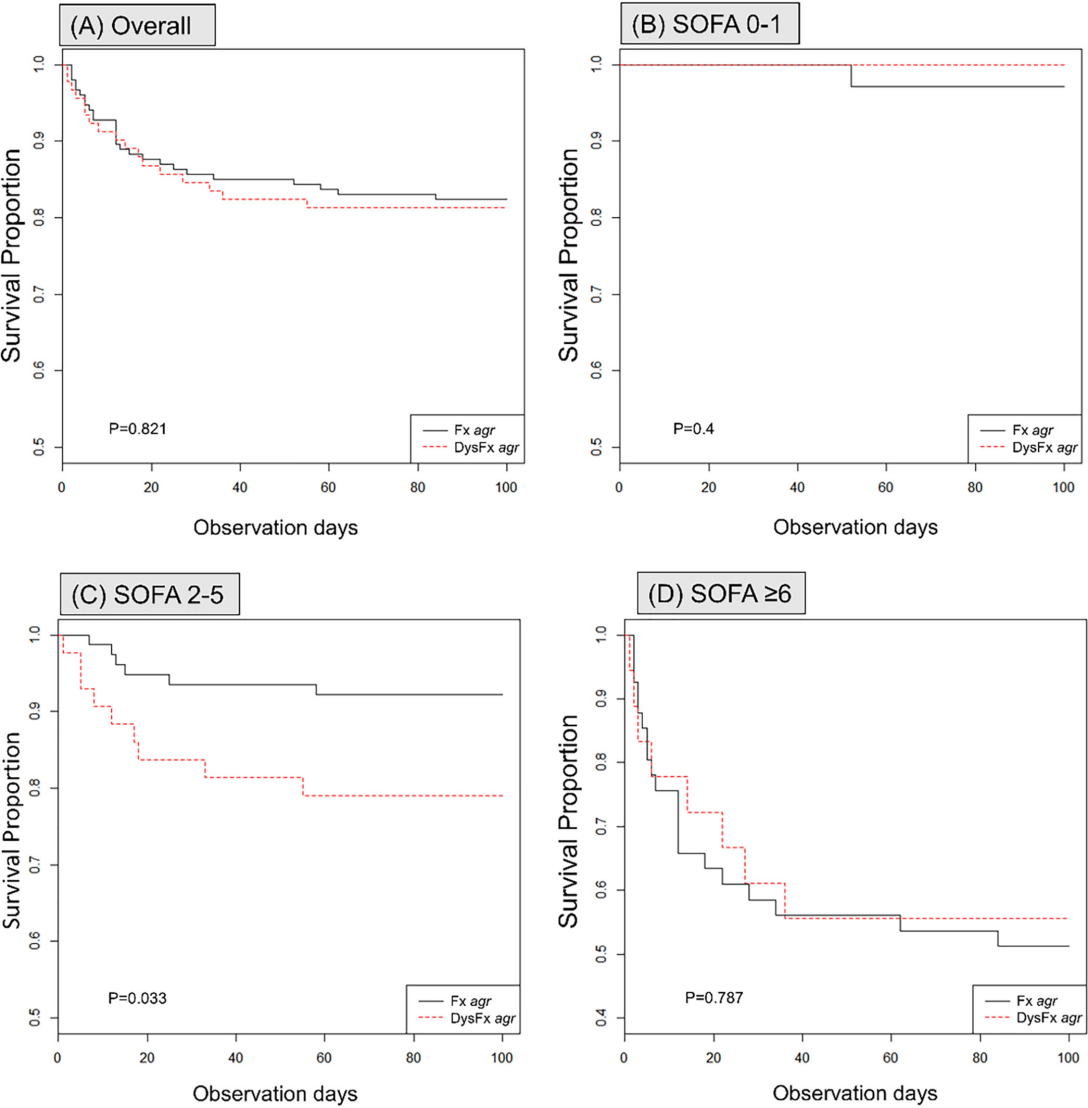
Kaplan-Meier survival curve for 90-day mortality of MSSA-B with dysfunctional *agr* group or functional *agr* group according to the SOFA score overall (A), SOFA scores of 0 to 1 (B), SOFA score of 2 to 5 (C), SOFA scores of ≥6 (D).

Kaplan-Meier analysis showed that the mortality of MSSA-B in the dysfunctional *agr* group was not significantly higher than that in the functional group (Fig. 1A; *P* = 0.82). In subgroup survival analysis with a SOFA score of 2 to 5, the mortality of MSSA-B was significantly higher in the dysfunctional *agr* group (Fig. 1C; *P* = 0.03) than in the functional *agr* group, while the mortality of MSSA-B was not significantly different between the two groups at SOFA scores of 0 to 1 (Fig. 1B; *P* = 0.4) and ≥6 (Fig. 1D; *P* = 0.79).

In the Cox proportional hazards regression analysis of the entire population (*n* = 244) with age and sites and severity of infection, comorbidity adjusted hazard ratio (aHR) of dysfunctional *agr* MSSA-B for 90-day mortality was 1.303 (95% confidence interval [CI], 0.698 to 2.436, *P* = 0.406). The Cox proportional hazards regression analysis for the subgroup of patients with MSSA-B at SOFA scores of 2 to 5 revealed that dysfunctional *agr* (aHR, 3.26; 95% CI, 1.05 to 10.12) and a high CCWI (aHR, 9.50; 95% CI, 1.01 to 89.84) were independently associated with mortality.

## DISCUSSION

S. aureus bacteremia (SAB) is a common bacterial infection but one of the most serious, with an associated mortality of about 20 to 30% despite advancing antibiotic treatments (3, 10, 12, 13). Risk factors affecting the high mortality include age, comorbid illness, the presence of metastatic foci, methicillin resistance, the severity of sepsis, and delayed appropriate antimicrobial therapy (3, 14, 15). S. aureus produces various potential virulence factors, which may cause poor clinical outcomes in SAB (3). Among these virulence factors, dysfunction of the *agr* locus has been studied frequently as a predictive factor of SAB, especially in MRSA.

In a recent meta-analysis, dysfunctional *agr* in invasive S. aureus infection was a significant risk factor for poor clinical outcomes, with the effect on the outcomes being more pronounced in invasive MRSA infection with dysfunctional *agr* than in that with functional *agr* (10). Contrastingly, invasive MSSA infection with dysfunctional *agr* was not associated with unfavorable outcomes and showed 30-day mortality lower than that with functional *agr* (10). However, the implications of *agr* dysfunction on the outcomes of invasive MSSA infection may not have been identified because of the insufficient number of the study subjects, the heterogeneity of the severity of infection, or the varied endpoints in previous studies (10). In this study, we evaluated the association between the outcomes of MSSA-B and *agr* functionality using stratified analysis to reduce the implications of the heterogeneity and severity of the infection. The findings of the present study provide useful information.

First, our study demonstrated that dysfunctional *agr* was associated with survival lower in patients with MSSA-B with a SOFA score of 2 to 5 than that in patients with other SOFA scores. As is generally known, the severity of disease at initial presentation is one of the strongest prognostic factors of SAB (3, 14, 16, 17). In accordance with previous studies, the 90-day mortality increased rapidly with increasing SOFA scores (1.5%, 12.5%, and 47.5% at SOFA scores 0 to 1, 2 to 5, and ≥6, respectively). We hypothesized that it would be difficult to assess the impact of other factors because of the impact of disease severity, which is the strongest risk factor. Therefore, we performed a stratified analysis using the SOFA score, which is useful for measuring disease severity and predicting the clinical outcomes (18). In the overall analysis of our data, there was no statistically significant difference in mortality between dysfunctional and functional *agr* groups. This finding is consistent with previous studies showing that MSSA-B with dysfunctional *agr* was not associated with higher mortality compared to that of MSSA-B with functional *agr* (10, 16, 19, 20). However, in patients with moderate illness at SOFA scores of 2 to 5, dysfunctional *agr* was an independent risk factor for mortality with MSSA-B. In contrast, patients with severe illness at a SOFA score of ≥6 had a high mortality rate, while patients with mild illness at SOFA scores of 0 to 1 showed a very low mortality rate regardless of the *agr* functionality. These findings suggest that the loss of *agr* function may enhance the adaptability of the causative organism for long-term survival in the host, contributing to chronic infection with increased long-term mortality, even in MSSA.

Second, our study demonstrated that approximately one-third of the patients with MSSA-B were infected with isolates with *agr* dysfunction. A considerable proportion of MSSA isolates possessed a dysfunctional *agr*, although this proportion is within the range reported by previous studies, from 8% to 48% of clinical MSSA isolates (16, 19–24). Previous MRSA studies have demonstrated that dysfunctional *agr* is associated with health care settings and multidrug resistance (11, 25–27). However, the strain included in our study was MSSA, and in most patients, the strain was neither multidrug-resistant nor hospital-acquired. Butterfield et al. (9) reported that prior beta-lactam and fluoroquinolone exposure are predictive factors of *agr* dysfunction. Therefore, the relatively high dysfunctional *agr* rate of MSSA in our study might be due to the relatively high consumption of antibiotics in Korea (28, 29).

Third, our study revealed that comorbidities, the severity of the infectious disease, and sites of infection were important factors affecting SAB outcomes. The risk factors associated with the 90-day mortality with MSSA-B included a high CCWI, a high SOFA score, and the occurrence of infective endocarditis, while the community-onset infection was negatively associated with mortality, which is consistent with previous studies (3, 14, 15, 30).

This study has several limitations. First, it was a single-center, retrospective study with a small sample size, and we cannot rule out the presence of unmeasured confounding factors. Second, data analyses were restricted to those with computerized medical records; some variables that may be associated with mortality were not available because of the lack of data. Third, we performed delta hemolysin assay using the isolates stored/frozen at −80°C. Freezing and long-term storage of the isolate might have affected the *agr* function of the isolates, although freezing is a very common method of preservation and storage of microorganisms (31).

In summary, *agr* dysfunctional MSSA-B was not uncommon. MSSA-B patients with moderate severity and SOFA scores between 2 to 5 and dysfunctional *agr* were associated with lower survival. The *agr* dysfunction in causative organisms may have a significant effect on the outcomes of patients with MSSA-B and MRSA-B. Dysfunction of *agr* might be useful as a biomarker for the outcomes of patients with MSSA bacteremia. Further studies are warranted to intensify our findings.

## MATERIALS AND METHODS

### Study design and population.

A retrospective cohort study was conducted to evaluate the comparative outcomes of MSSA-B with dysfunctional versus functional *agr* groups at a 1,300-bedded tertiary care hospital in Korea from June 2014 to June 2019. Patients (≥15 years of age) with MSSA-B were enrolled; patients with polymicrobial infection or suspected culture contamination were excluded. Only the first bloodstream culture isolate from each patient was included in the study. We assigned the patients with MSSA-B to dysfunctional or functional *agr* groups according to the *agr* functionality of causative MSSA isolates.

The study protocol was approved by the institutional review board (IRB) of Pusan National University Hospital (IRB no. H-2010-002-095), and the requirement of informed consent was waived.

### Data collection and definitions.

We collected demographic data, clinical information, and antimicrobial susceptibility data of the participants retrospectively by reviewing the medical records. Infection was classified as community-onset if an S. aureus-positive specimen was obtained within 72 h of hospitalization (32). Infections were classified based on the site of infection as central line-associated bloodstream infection, infective endocarditis, pneumonia, osteomyelitis, septic arthritis, skin and soft tissue infections, deep-seated abscess, and unknown primary focus (33). The severity of the comorbidity was measured using the CCWI (34), while the severity of the bacteremia was measured using the sequential organ failure assessment (SOFA) score (18). Antimicrobial therapy was considered appropriate if the isolate was susceptible to the antibiotic chosen *in vitro*. Empirical and definitive antibiotics were defined as antibiotics administered within the first 48 h and those used after a report of antibiotic susceptibility of the isolates, respectively (35). Persistent bacteremia was defined as the first negative blood culture occurring >7 days after the first positive blood culture with appropriate antibiotic therapy (36).

### Microbiological analyses.

Microbiological analyses were conducted using the isolates that were collected from patients and stored at −80°C. The functionality of the *agr* locus was measured through δ-hemolysin expression assays using the S. aureus strain RN4220 as an indicator, and the absence of or barely detectable synergistic hemolysis was defined as *agr* dysfunction (4). To determine the *agr* group genotype, *agr* group-specific multiplex PCR was performed using primers that have been described previously (37). The cefazolin inoculum effect was measured by comparing the MICs of the high inoculum (∼5 × 10^7^ CFU/ml) with those of the standard inoculum (∼5 × 10^5^ CFU/ml), as described previously (38, 39). Cefazolin inoculum effect was defined as an increase in MICs to ≥16 μg/ml at high inoculum from a susceptible range of MIC at standard inoculum (33).

### Main outcome.

The main outcome of our study was the 90-day mortality rate. We also compared the rates of persistent bacteremia, 7-day mortality, and 30-day mortality. To analyze the difference in the effect of dysfunctional *agr* on the outcome of MSSA-B according to the severity of the initial infectious disease, we performed a stratified analysis according to the SOFA score (0 to 1, 2 to 5, ≥6).

### Statistical analysis.

R (version 3.3.2; R Foundation for Statistical Computing, Vienna, Austria) was used for all statistical analyses. Categorical variables were compared using Pearson’s chi-square test or Fisher’s exact test, and noncategorical variables were tested using the Mann-Whitney U test or Kruskal-Wallis test. Logistic regression analysis was used to determine the risk factors for 90-day mortality. Variables with a *P* value of <0.2 in bivariate analysis were included in a multivariate logistic regression model, and *agr* functionality and cefazolin inoculum effect were forced separately into the model to estimate their clinical implications. The Kaplan-Meier method was used to determine differences in survival between the dysfunctional and functional *agr* groups according to the initial SOFA score. Cox proportional hazards regression analysis was used to examine the association between *agr* functionality and mortality, where patients were censored if death occurred within 90 days after the first positive blood culture result was collected. The initial SOFA score (0 to 1, 2 to 5, ≥6) was stratified by *agr* functionality (functional and dysfunctional) during subgroup analysis. CCWI and definitive antibiotics were incorporated into the models as predictors of mortality. aHRs and 95% CIs were calculated using multivariate modeling. All tests of significance were 2-tailed, and the results were considered statistically significant at *P* < 0.05.
